# Supplementary material for: Emergency department attendance by callers to NHS111 who end the call prior to triage: A time-to-event-analysis
Source: PLoS One. 2026 Apr 21;21(4):e0346969. doi: 10.1371/journal.pone.0346969 (PMC13098975; doi:10.1371/journal.pone.0346969)
Supplement: S1 Table — (PDF) [file pone.0346969.s001.pdf]

S1-Table: Modification to the original O'Keeffe et al non-avoidable Emergency Department admission criteria

**Disposition code**	**Potentially avoidable**
ED Treatment complete	Yes
Admitted as inpatient	No
Streamed to GP / primary care	Yes
Left after assessment before treatment	Yes
Left before initial assessment	Yes
Left after assessment other	Yes
Streamed to Urgent Care Centre	Yes
Streamed to ophthalmology service	Yes
Died in the Emergency Care facility	No
Streamed to Amb Care service	No
Streamed to mental health service	Yes
Streamed to Emergency Department	No
Streamed to dental service	Yes
Dead on Arrival	No
Discharged with Consent	Yes
Streamed to pharmacy service	Yes
Streamed to falls service	No
Streamed to frailty service	No
