# Supplementary material for: Emergency department attendance by callers to NHS111 who end the call prior to triage: A time-to-event-analysis
Source: PLoS One. 2026 Apr 21;21(4):e0346969. doi: 10.1371/journal.pone.0346969 (PMC13098975; doi:10.1371/journal.pone.0346969)
Supplement: S1 File — (PDF) [file pone.0346969.s002.pdf]

S1-File:: Summary of final model for the EXPECT study

Kaplan-Meier plots

Simple model looking at cohort only

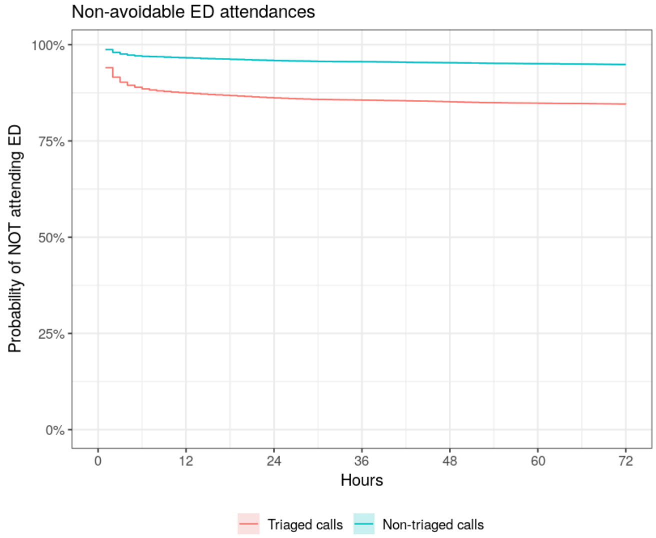

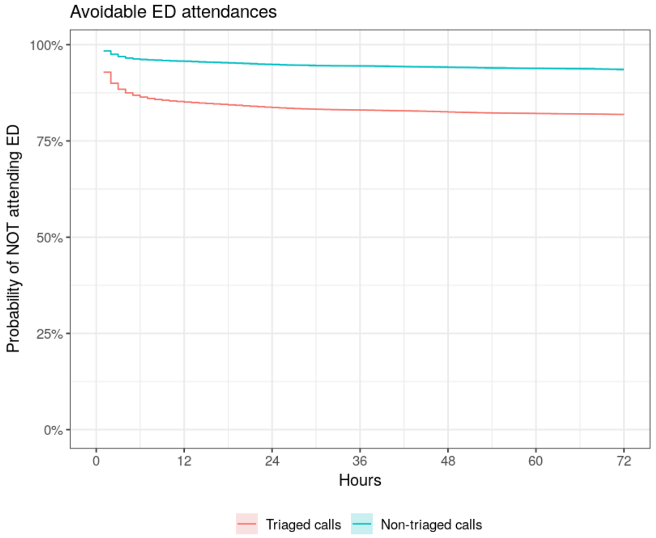

Log-log plot

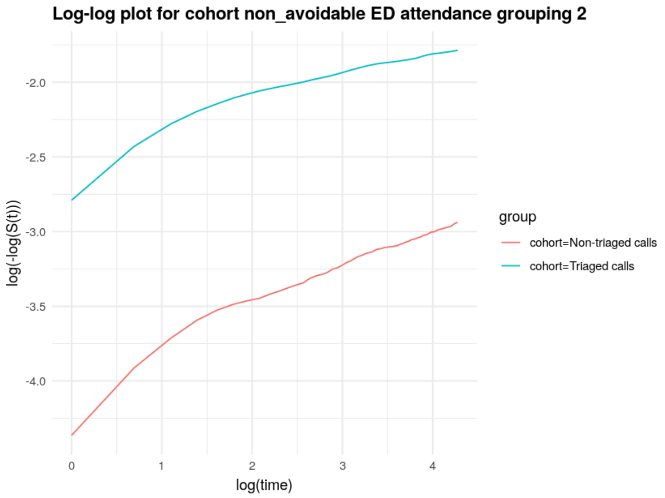

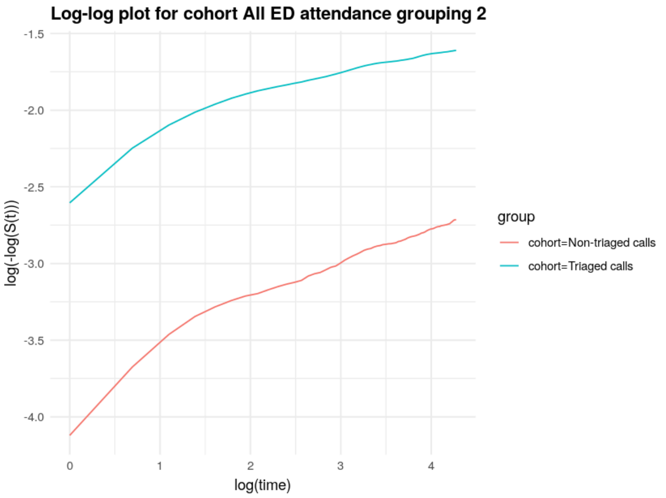

Cox regression

Non-avoidable ED attendances

A tibble: 12 × 8

term	estimate	std.error	robust.se	statistic	p.value	conf.low	conf.high
<chr>	<dbl>	<dbl>	<dbl>	<dbl>	<dbl>	<dbl>	<dbl>

term	estimate	std.error	robust.se	statistic	p.value	conf.low	conf.high
<chr>	<dbl>	<dbl>	<dbl>	<dbl>	<dbl>	<dbl>	<dbl>
cohortNon-triaged calls	0.32	0.03	0.03	-33.29	0.00	0.30	0.34
age	1.01	0.00	0.00	41.59	0.00	1.01	1.01
sexmale	0.93	0.01	0.02	-5.03	0.00	0.90	0.95
imd_quintile2	0.96	0.02	0.02	-2.23	0.03	0.92	0.99
imd_quintile3	0.90	0.02	0.02	-4.51	0.00	0.86	0.94
imd_quintile4	0.86	0.02	0.03	-5.79	0.00	0.82	0.90
imd_quintile5	0.75	0.03	0.03	-9.80	0.00	0.70	0.79
ethnicity_simpleAsian or Asian British	0.98	0.02	0.02	-0.88	0.38	0.95	1.02
ethnicity_simpleBlack or African or Caribbean or Black British	0.81	0.06	0.06	-3.34	0.00	0.72	0.92
ethnicity_simpleMixed multiple ethnic groups	0.96	0.06	0.11	-0.42	0.67	0.77	1.18
ethnicity_simpleOther ethnic group	0.79	0.06	0.07	-3.37	0.00	0.69	0.91
ethnicity_simpleUnknown/Refuse to say	0.86	0.02	0.02	-8.02	0.00	0.83	0.89

All ED attendances

A tibble: 12 × 8

term	estimate	std.error	robust.se	statistic	p.value	conf.low	conf.high
<chr>	<dbl>	<dbl>	<dbl>	<dbl>	<dbl>	<dbl>	<dbl>
cohortNon-triaged calls	0.33	0.03	0.03	-35.87	0.00	0.31	0.35
age	1.01	0.00	0.00	32.92	0.00	1.01	1.01
sexmale	0.90	0.01	0.01	-7.66	0.00	0.87	0.92
imd_quintile2	0.96	0.02	0.02	-2.48	0.01	0.92	0.99
imd_quintile3	0.89	0.02	0.02	-5.00	0.00	0.85	0.93
imd_quintile4	0.88	0.02	0.03	-4.95	0.00	0.84	0.93
imd_quintile5	0.77	0.02	0.03	-9.44	0.00	0.73	0.81
ethnicity_simpleAsian or Asian British	1.02	0.02	0.02	0.90	0.37	0.98	1.05
ethnicity_simpleBlack or African or Caribbean or Black British	0.80	0.05	0.06	-3.69	0.00	0.72	0.90
ethnicity_simpleMixed multiple ethnic groups	0.96	0.05	0.09	-0.42	0.67	0.80	1.16
ethnicity_simpleOther ethnic group	0.87	0.05	0.06	-2.16	0.03	0.77	0.99
ethnicity_simpleUnknown/Refuse to say	0.85	0.01	0.02	-9.29	0.00	0.82	0.88

Assessing Goodness-of-Fit using residuals

Martingale residuals

Non-avoidable ED attendance

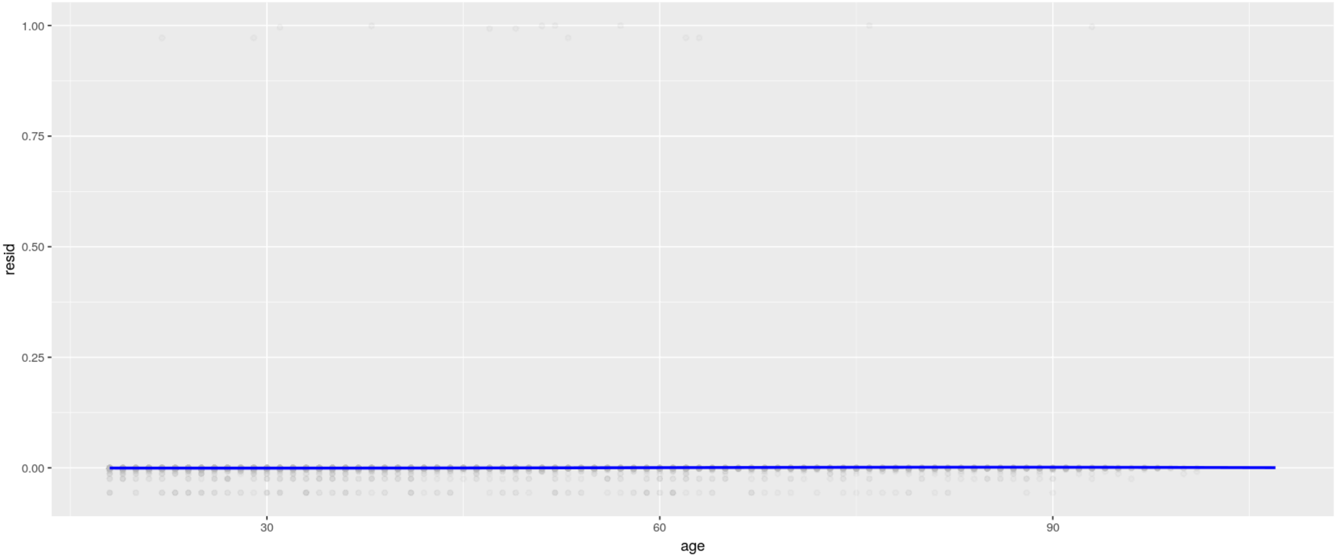

All ED attendances

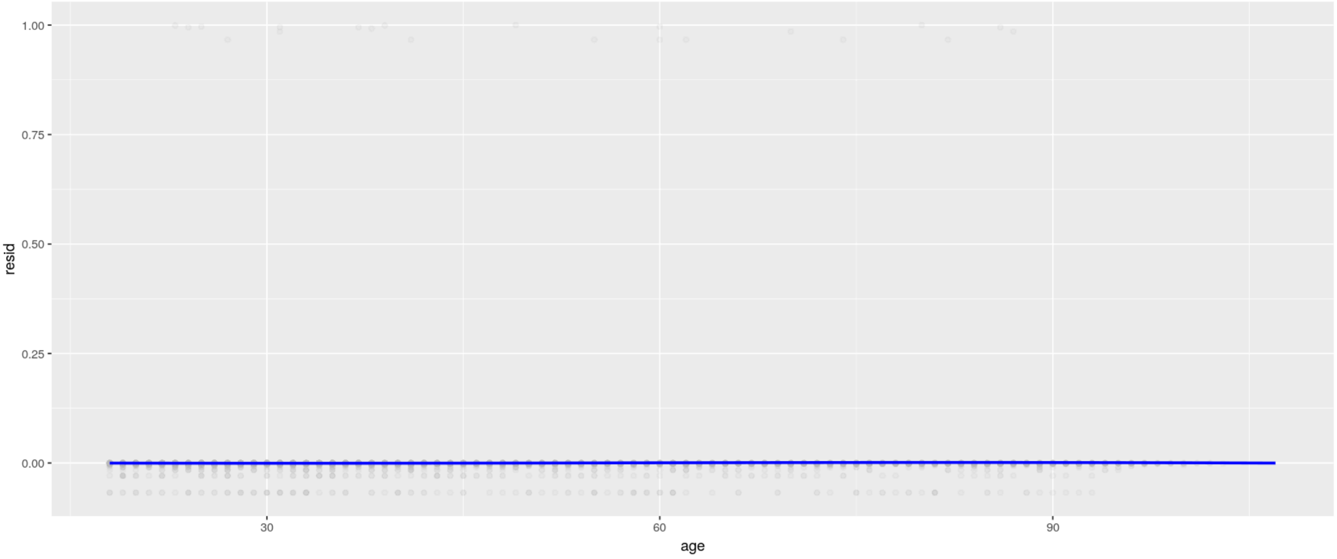

Checking the proportional hazards assumption

Log-log plots

Non-avoidable ED attendances

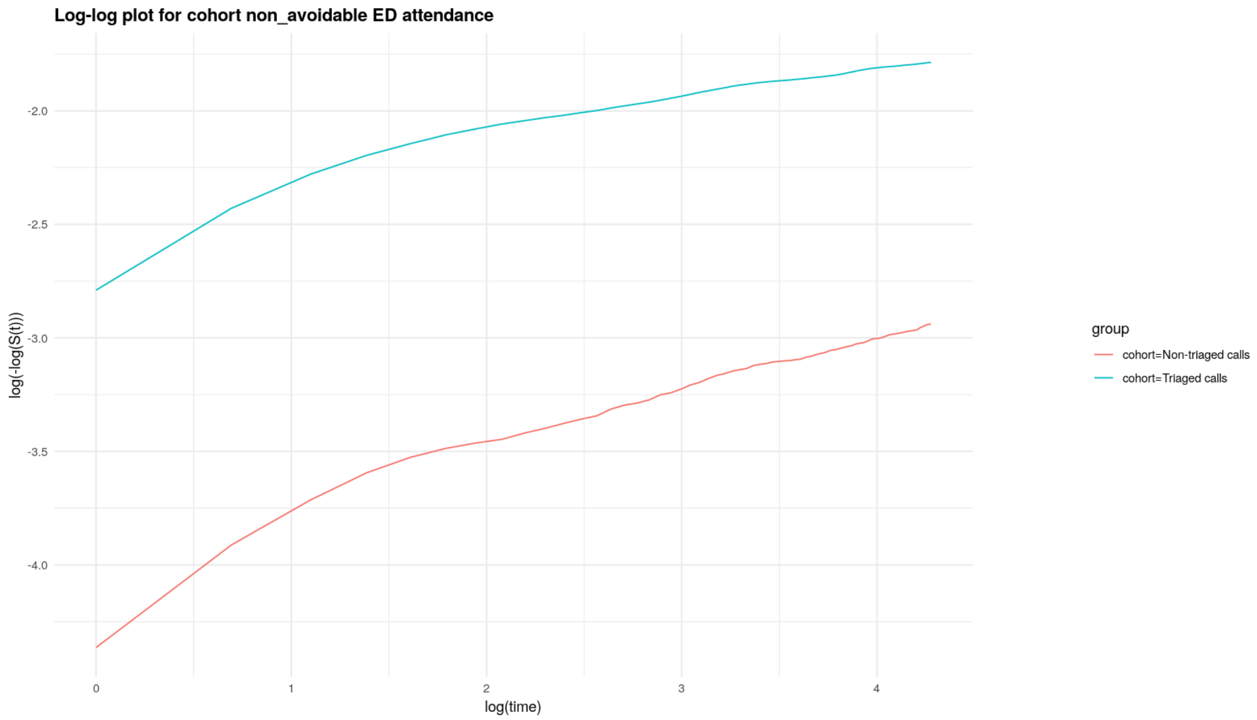

Log-log plot for cohort non_avoidable ED attendance
-2.0
-2.5
-3.0
-3.5
-4.0
log(-log(S(t)))
0
1
2
3
4
log(time)
group
cohort=Non-triaged calls
cohort=Triaged calls

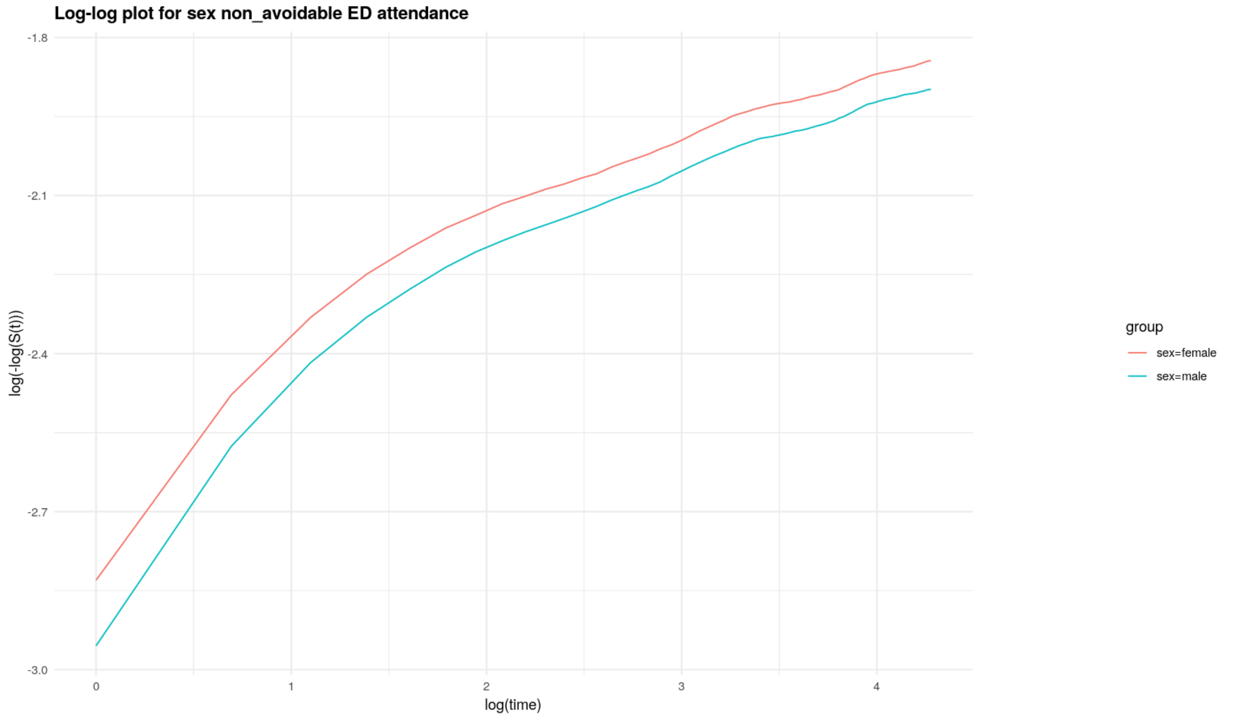

Log-log plot for sex non_avoidable ED attendance
-1.8
-2.1
-2.4
-2.7
-3.0
log(-log(S(t)))
0
1
2
3
4
log(time)
group
sex=female
sex=male

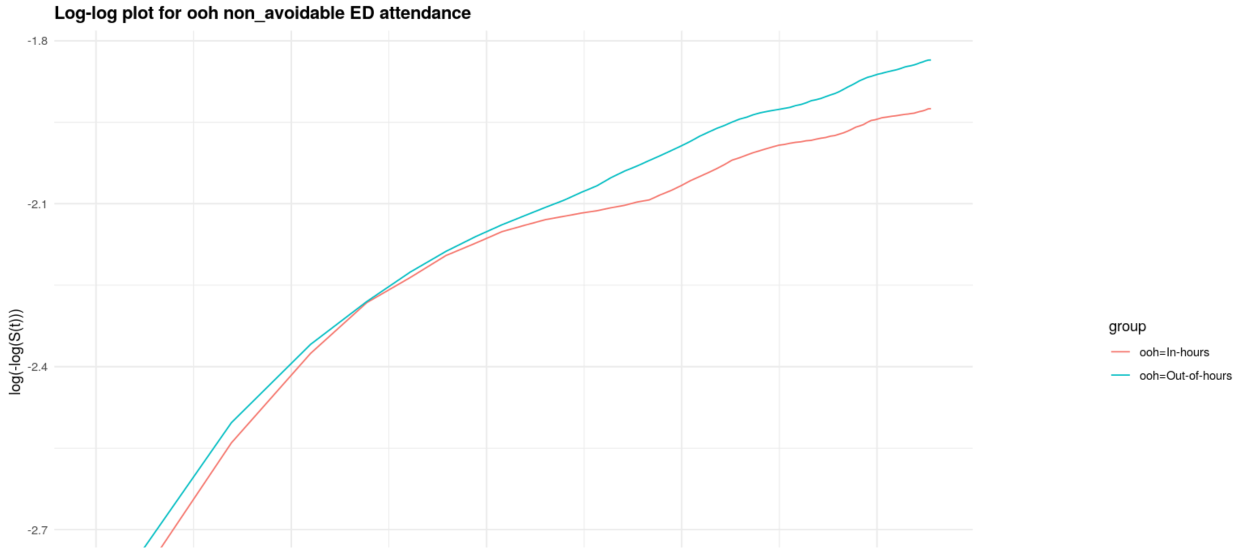

Log-log plot for ooh non_avoidable ED attendance
-1.8
-2.1
-2.4
-2.7
log(-log(S(t)))
group
ooh=In-hours
ooh=Out-of-hours

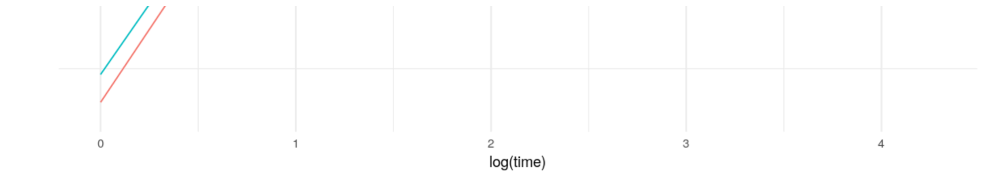

Log-log plot for imd_quintile non_avoidable ED attendance

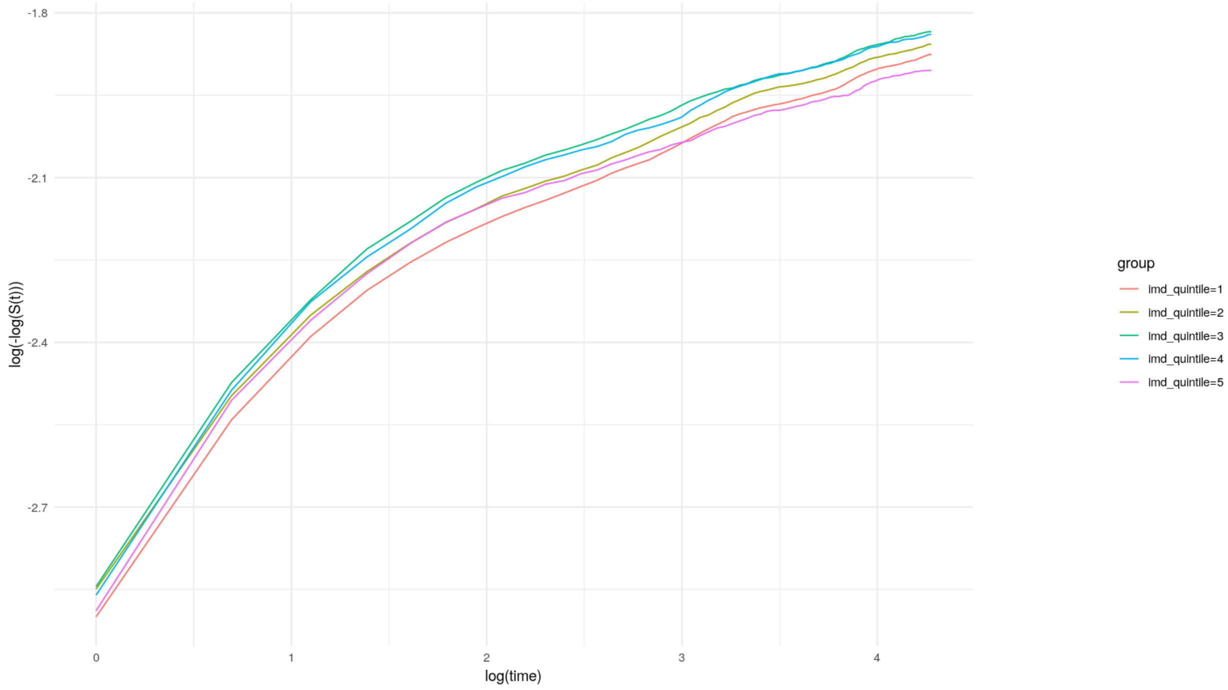

Log-log plot for ethnicity_simple non_avoidable ED attendance

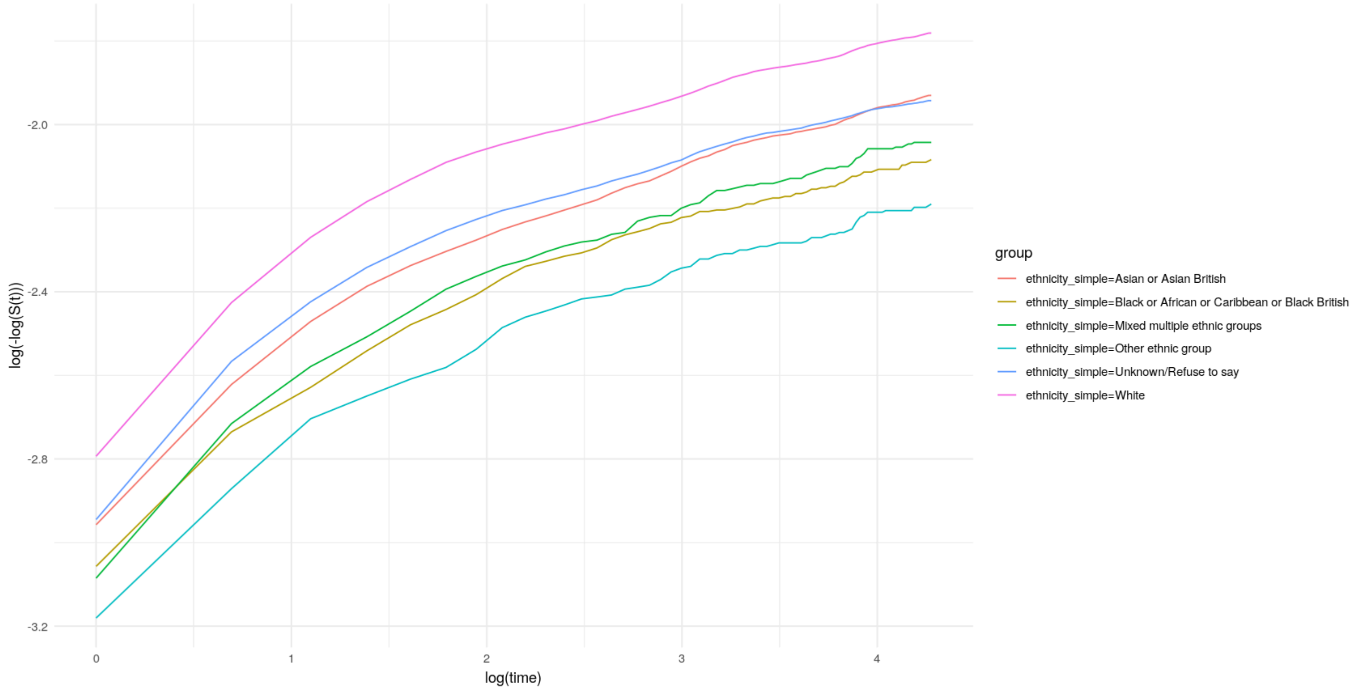

All ED attendances

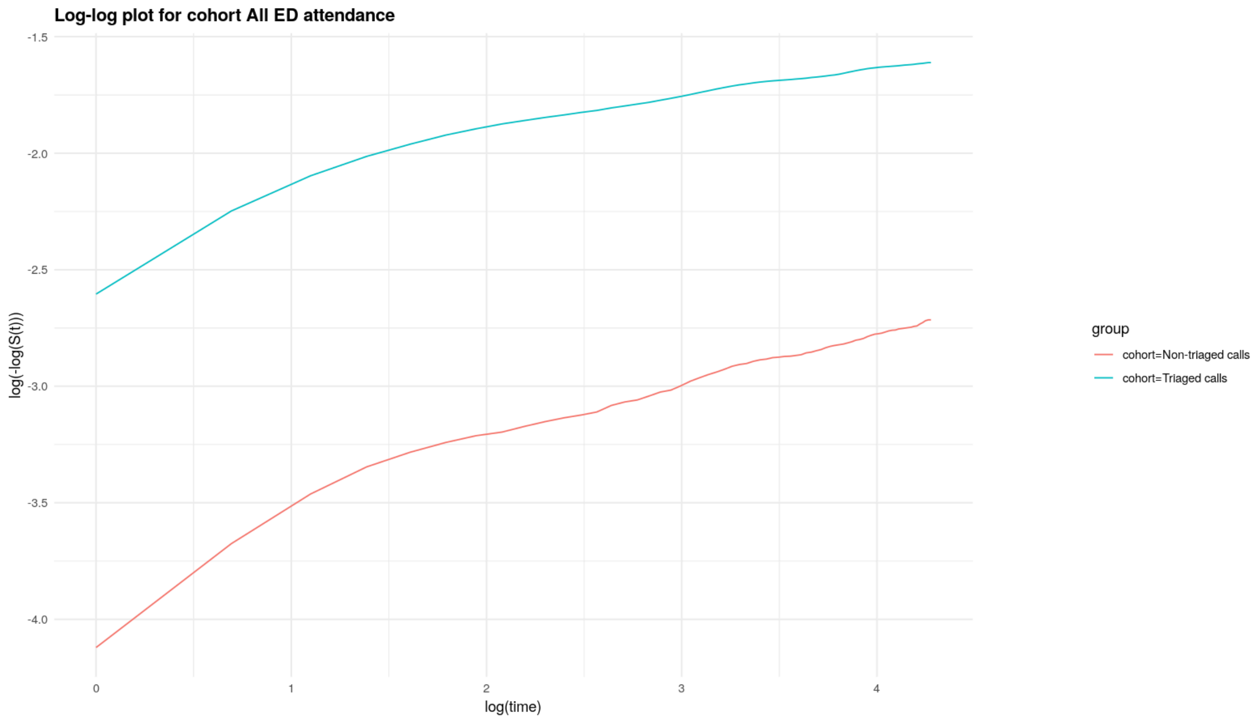
Log-log plot for cohort All ED attendance
-1.5
-2.0
-2.5
-3.0
-3.5
-4.0
log(-log(S(t)))
0
1
2
3
4
log(time)
group
cohort=Non-triaged calls
cohort=Triaged calls

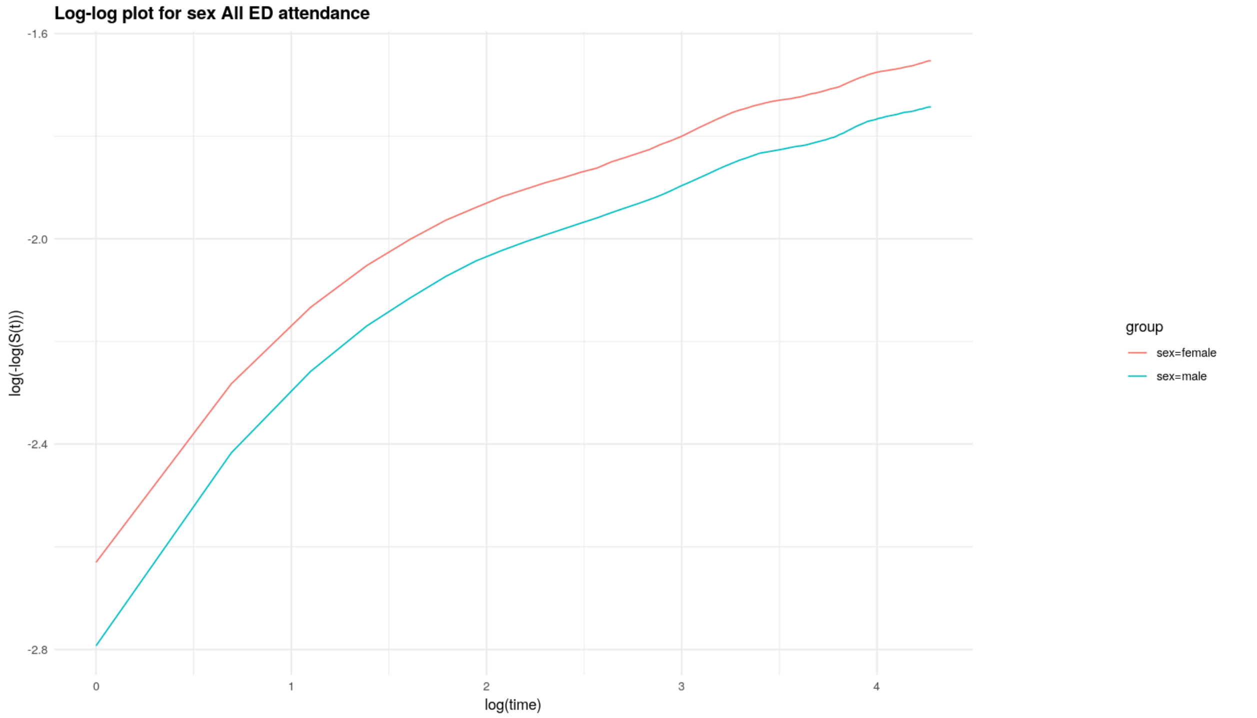
Log-log plot for sex All ED attendance
-1.6
-2.0
-2.4
-2.8
log(-log(S(t)))
0
1
2
3
4
log(time)
group
sex=female
sex=male

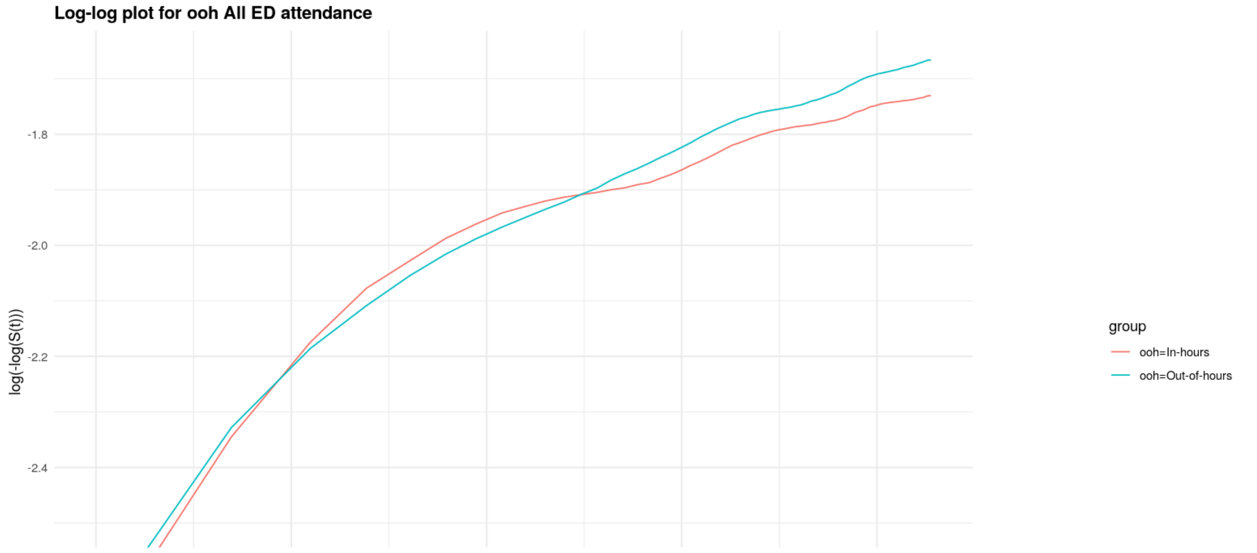
Log-log plot for ooh All ED attendance
-1.8
-2.0
-2.2
-2.4
log(-log(S(t)))
group
ooh=In-hours
ooh=Out-of-hours

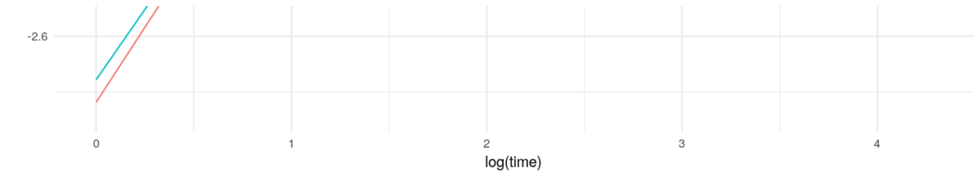

Log-log plot for imd_quintile All ED attendance

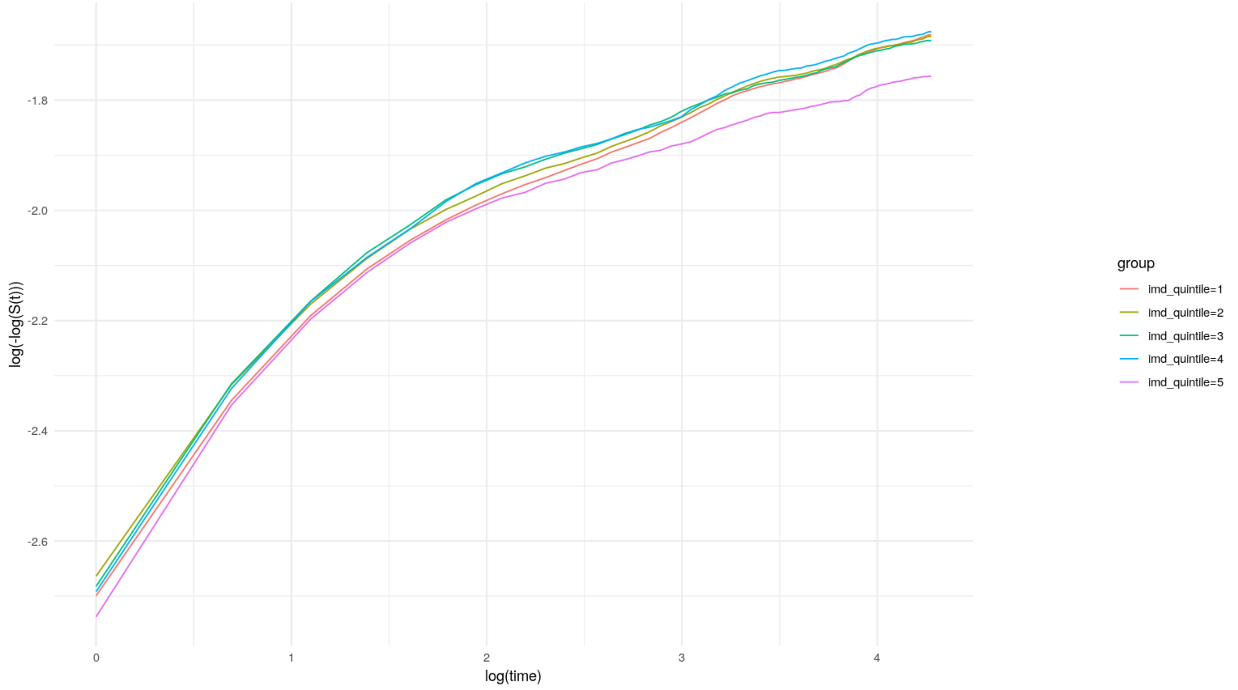

Log-log plot for ethnicity_simple All ED attendance

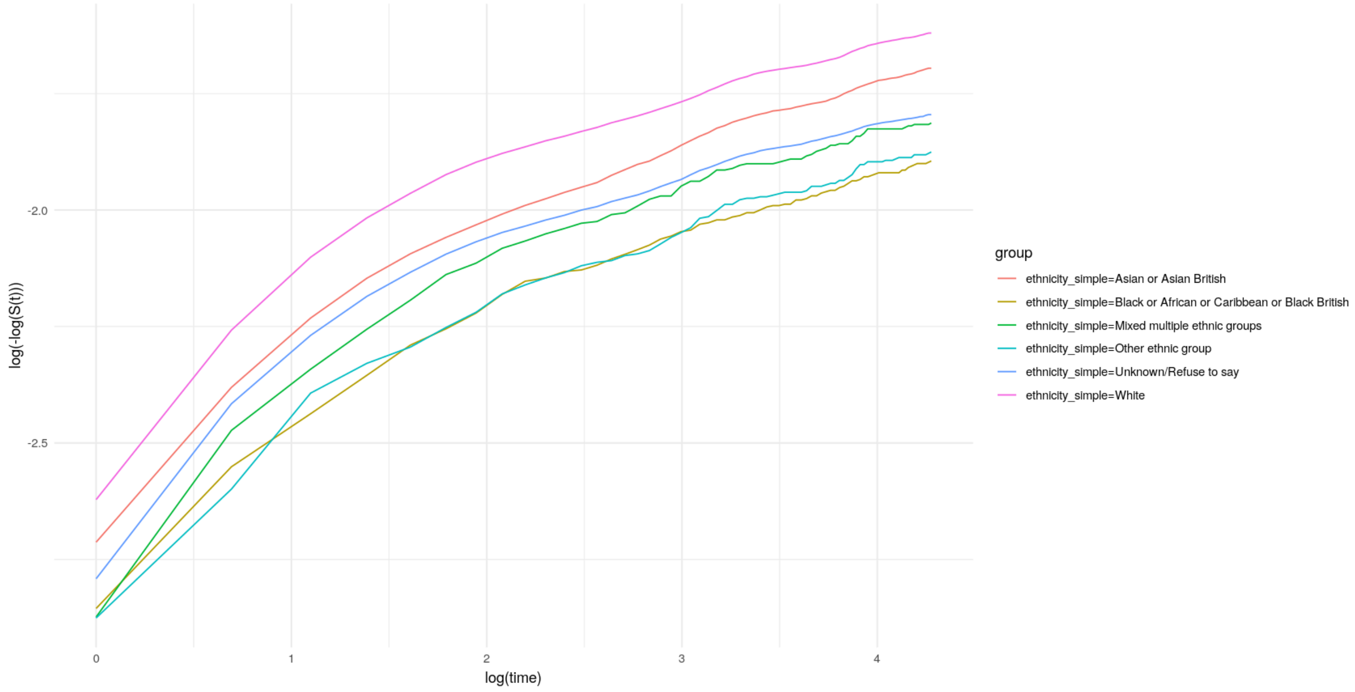

Non-avoidable ED attendances

```
                  chisq df       p
cohort           105.27  1 < 2e-16
age                3.58  1   0.058
sex               17.15  1 3.5e-05
imd_quintile      11.18  4   0.025
```

```
ethnicity_simple  12.20  5   0.032
GLOBAL           141.11 12 < 2e-16
```

All ED attendances

```
                   chisq df      p
cohort           125.059  1 <2e-16
age                0.529  1 0.4670
sex               21.805  1  3e-06
imd_quintile      17.216  4 0.0018
ethnicity_simple  14.833  5 0.0111
GLOBAL           172.669 12 <2e-16
```

Schoenfeld residuals

Non-avoidable ED attendances

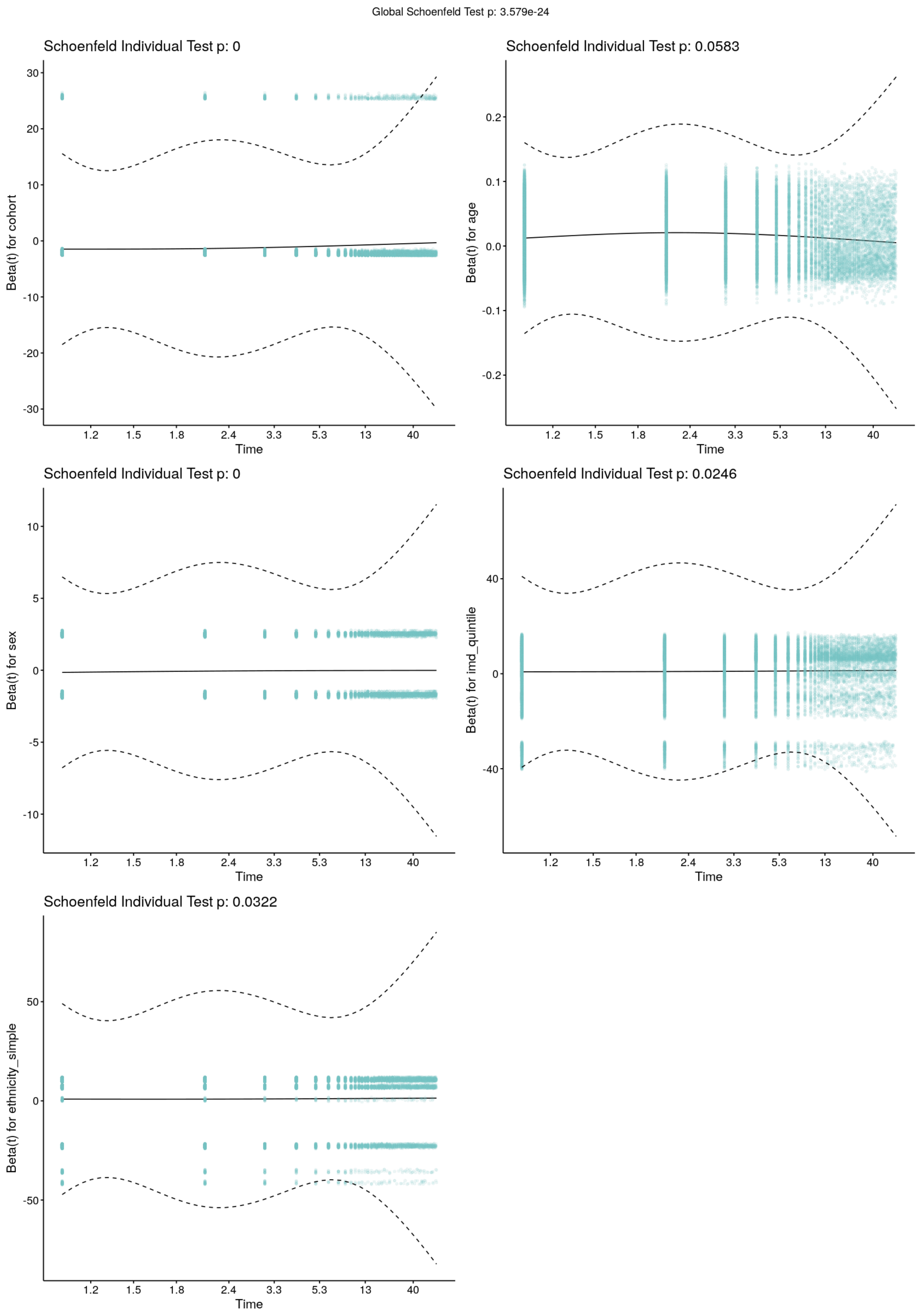

All ED attendances

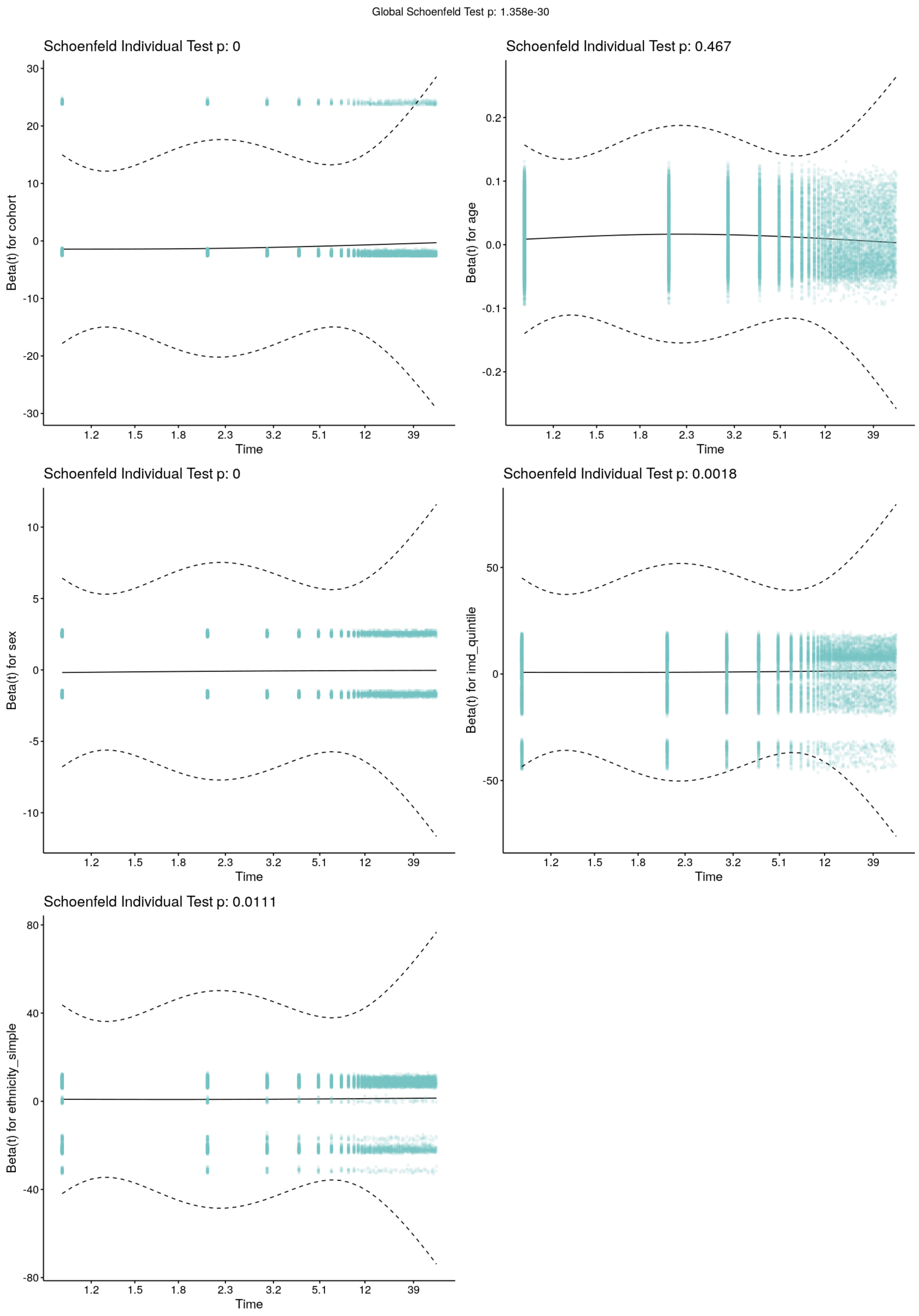
Global Schoenfeld Test p: 1.358e-30
Schoenfeld Individual Test p: 0
Beta(t) for cohort
30
20
10
0
-10
-20
-30
1.2
1.5
1.8
2.3
3.2
5.1
12
39
Time
Schoenfeld Individual Test p: 0.467
Beta(t) for age
0.2
0.1
0.0
-0.1
-0.2
Time
Schoenfeld Individual Test p: 0
Beta(t) for sex
10
5
0
-5
-10
Time
Schoenfeld Individual Test p: 0.0018
Beta(t) for imd_quintile
50
0
-50
Time
Schoenfeld Individual Test p: 0.0111
Beta(t) for ethnicity_simple
80
40
0
-40
-80
Time
