# Supplementary material for: Emergency department attendance by callers to NHS111 who end the call prior to triage: A time-to-event-analysis
Source: PLoS One. 2026 Apr 21;21(4):e0346969. doi: 10.1371/journal.pone.0346969 (PMC13098975; doi:10.1371/journal.pone.0346969)
Supplement: Attachment — Submitted filename: Authors-response-to-reviewers-comments.docx [file pone.0346969.s004.docx]

| **Reviewer comment** | **Author’s response** |
| --- | --- |
| The title should be revised to ensure it is more scientific and accurately reflects the study's focus and outcomes. | Title updated |
| The methodology section needs further details on the study design, including the specific variables assessed and data collection methods. Additionally, the statistical methods and software used should be explicitly mentioned. | Addition of the use of R and specific time-to-event analysis tools added.  Specific variables are stated in the primary analysis section of the methods. |
| The statistical analysis requires improvement. The authors should apply the Chi-square (χ²) test to analyze the association between patient characteristics and call outcomes. | It is the view of the statisticians that Chi-square tests i.e. univariate testing are not necessary or informative since the table is describing the cohort and important covariates are already controlled in the Cox analysis. |
| Table 1 should include call duration as either means with standard deviations or categorized values for better interpretation. | Call duration was not available in the data and so has not been presented. The other continuous variables have been presented as median and inter-quartile ranges given that the data was not normally distributed and mean/standard deviation was not appropriate. |
| The manuscript should ensure clarity in terminology and abbreviations by defining IMD in the key notes of Table 1 to enhance reader understanding. | The abbreviations used in the table were provided in full in the text prior to table 1. However, IMD and ED have been writing in full in table 1 to improve clarity as requested. |
| The results section should provide a more detailed explanation of the findings, ensuring they are well-supported by data and statistical analysis. | We are struggling to know what is being requested here without repeating the contents of the tables. We would appreciate some specific advice as to what the reviewers feel is missing. |
| The discussion and recommendations should include practical suggestions on improving patient outcomes based on the study’s findings. | Additional paragraph added to the Discussion section to address this. |
| The ethics and acknowledgments sections should explicitly state ethical considerations and disclose financial support sources, if applicable, to maintain transparency. | Ethics section updated |
| Please ensure that your manuscript meets PLOS ONE's style requirements, including those for file naming. The PLOS ONE style templates can be found at | Requirements reviewed and manuscript updated to conform. |
| 'Please provide additional details regarding participant consent. In the ethics statement in the Methods and online submission information, please ensure that you have specified (1) whether consent was informed and (2) what type you obtained (for instance, written or verbal, and if verbal, how it was documented and witnessed). If your study included minors, state whether you obtained consent from parents or guardians. If the need for consent was waived by the ethics committee, please include this information. | Ethics section updated to highlight that the research database already has the necessary ethical approvals for the use of patient data for research purposed without the need for individual patient consent.  Note that this study only involved adults. |
| If you are reporting a retrospective study of medical records or archived samples, please ensure that you have discussed whether all data were fully anonymized before you accessed them and/or whether the IRB or ethics committee waived the requirement for informed consent. If patients provided informed written consent to have data from their medical records used in research, please include this information. | The Data section explains this. Ethics section has been updated. |
| 3. Thank you for stating the following financial disclosure: [This report is independent research funded by the National Institute for Health and Care Research, Yorkshire and Humber Applied Research Collaborations NIHR200166. This study is also based on data from Connected Bradford (REC 18/YH/0200 & 22/EM/0127). The data is provided by the citizens of Bradford and district, and collected by the NHS, DfE and other organisations as part of their care and support. The views expressed in this publication are those of the author(s) and not necessarily those of the NHS, the National Institute for Health and Care Research or the Department of Health and Social Care.]. Please state what role the funders took in the study. If the funders had no role, please state: "The funders had no role in study design, data collection and analysis, decision to publish, or preparation of the manuscript." If this statement is not correct you must amend it as needed. Please include this amended Role of Funder statement in your cover letter; we will change the online submission form on your behalf. | Statement updated as requested. |
| 4. We note that you have indicated that there are restrictions to data sharing for this study. For studies involving human research participant data or other sensitive data, we encourage authors to share de-identified or anonymized data. However, when data cannot be publicly shared for ethical reasons, we allow authors to make their data sets available upon request. For information on unacceptable data access restrictions, please see http://journals.plos.org/plosone/s/data-availability#loc-unacceptable-data-access-restrictions.  Before we proceed with your manuscript, please address the following prompts:  **a) If there are ethical or legal restrictions on sharing a de-identified data set, please explain them in detail (e.g., data contain potentially identifying or sensitive patient information, data are owned by a third-party organization, etc.) and who has imposed them (e.g., a Research Ethics Committee or Institutional Review Board, etc.). Please also provide contact information for a data access committee, ethics committee, or other institutional body to which data requests may be sent.**  b) If there are no restrictions, please upload the minimal anonymized data set necessary to replicate your study findings to a stable, public repository and provide us with the relevant URLs, DOIs, or accession numbers. Please see http://www.bmj.com/content/340/bmj.c181.long for guidelines on how to de-identify and prepare clinical data for publication. For a list of recommended repositories, please see https://journals.plos.org/plosone/s/recommended-repositories. You also have the option of uploading the data as Supporting Information files, but we would recommend depositing data directly to a data repository if possible. | Data sharing statement has been updated on the portal. |
| Please include a caption for figure 1. | The caption for Figure 1 is on page 11 of the manuscript |
