# Supplementary material for: Emergency department attendance by callers to NHS111 who end the call prior to triage: A time-to-event-analysis
Source: PLoS One. 2026 Apr 21;21(4):e0346969. doi: 10.1371/journal.pone.0346969 (PMC13098975; doi:10.1371/journal.pone.0346969)
Supplement: Attachment — Submitted filename: Authors-response-to-reviewers-questions.docx [file pone.0346969.s005.docx]

| **Reviewer comment** | **Author’s response** |
| --- | --- |
| 2. Is the manuscript technically sound, and do the data support the conclusions?  Reviewer #2: Partly | We have fully addressed the reviewer’s comments in the previous revision. If the reviewer can provide comprehensive and verbose feedback, then we would be happy to address any outstanding issues. |
| 3. Has the statistical analysis been performed appropriately and rigorously?  Reviewer #2: No | Two of the authors are professional statisticians and are satisfied that within the limitations of the observational data utilised for the study, the statistical analysis has been performed appropriate and rigorously. |
| Due to the review, changes in the text of the article have not been highlighted. | I include this for the attention of the editorial team since it would be helpful for reviewer’s to see the tracked change submission we provided |
| During the review, the acknowledgements and thanks have been specified at the end. | Noted. |
| The statement of the ethics committee has been included. | Noted. |
| The title has been revised. | Noted. |
| References should be included in the journal according to the journal's guidelines. | References updated as requested. |
| The discussion of this article can better review the studies | We have deliberately not done this since the studies are only tangentially related to the current study, and we did not want to bring any further attention to them. As we state in the manuscript, as far as we are aware, there is no research specifically looking at addressing our research question. |
|  |  |
