# Supplementary material for: Emergency department attendance by callers to NHS111 who end the call prior to triage: A time-to-event-analysis
Source: PLoS One. 2026 Apr 21;21(4):e0346969. doi: 10.1371/journal.pone.0346969 (PMC13098975; doi:10.1371/journal.pone.0346969)
Supplement: Attachment — Submitted filename: Authors-response-to-reviewers-questions_auresp_3.docx [file pone.0346969.s006.docx]

| **Reviewer comment** | **Author’s response** |
| --- | --- |
| The introduction could be better explained. During the review, the necessity of this study and the innovative aspects could play a role in highlighting the objectives of this study. | We feel that the current background section is appropriate and does not delay the reader from getting ‘into’ the paper. |
| Introduction:The introduction is useful and well written. It provides clear context on NHS 111 and explains why understanding call abandonment is an important area for research. The topic is timely and relevant to health service policy and patient safety. | Thank you. |
| In the case of the first study Patient compliance with NHS 111 advice: Analysis of adult call and ED attendance data 2013–2017, could it be similar to your study? | No, this study only examined successfully triaged calls and did not contain any follow-up involving primary care services. |
| Considering the authors' response that you mentioned a limited study in the discussion. Using similar studies can address the necessity of this research and the study gap, which plays an important role in explaining your study. | There are no other comparable studies, which made the discussion more challenging with respect to its context in the wider literature. |
| b.Remove or substantially revise the comparison with patients who leave the emergency department without being seen. These represent a different decision-making context and do not provide a valid analogy for telephone triage abandonment. | Removed the comparison as suggested. |
| Replace any causal language with associational phrasing. For example, instead of stating that triaging “supports patients to seek appropriate help more quickly”, state that triaging “was associated with shorter times to emergency department attendance”. | Thank you for the suggested wording change. Manuscript updated. |
| Correct the typographical error “whot” to “who”. | Spelling corrected. |
| End the abstract with a balanced statement acknowledging that the study design does not allow causal inference. | We have changed the language of the final statement that should make it clear to the reader that the results are not based on causal inference. However, we had added a specific sentence in the limitations section to reflect this suggestion. |
| The methods section is generally well described and demonstrates thoughtful use of the Connected Bradford dataset. The inclusion of linked data sources is a major strength. The overall structure is logical, and the statistical approach is appropriate for the research question. | Thank you. |
| Stats: Justify the choice of a seventy-two-hour window for emergency department attendance and a thirty-second threshold for defining an abandoned call. These thresholds appear arbitrary and may influence the observed association | 72 hours was deemed by local expert opinion to be a reasonable time period to have some confidence that the reason for the 111 call and ED attendance were related. The greater the time period, the more tenuous the relationship was thought to be.  The 30 second threshold was chosen as NHS England differentiates between calls that end at 30 seconds or less in reporting requirements for NHS 111 service performance. In addition, calls lasting less than 30 seconds are more likely to be made in error in our view. |
| a. Reframe all causal statements as associations. For instance, replace “triaging helps patients to seek appropriate care sooner” with “triaging was associated with earlier emergency department attendance”. This will ensure the interpretation remains aligned with the observational nature of the study. | Manuscript updated. |
| Limitations: Mention that the study period included times of exceptional NHS strain, including winter pressures and post-pandemic recovery, which may have affected both call volumes and caller behaviour. | We disagree. The time period of this study (and indeed up to the present day) suggests that the current volume and state of the NHS is the ‘new’ normal. As such, we think our results are representative of the post-pandemic state of the NHS, which is likely to remain for some time to come. |
| I also believe that a brief statement about future research should be included within the conclusion. The current ending summarises the findings well but would benefit from a forward-looking sentence that highlights the next steps. I suggest adding a line such as:  “Future research should explore why some callers abandon calls, whether their conditions truly require emergency care, and how call-handling processes can be optimised to ensure equitable and timely access to appropriate services.” | Thank you, manuscript updated with the suggestion. |
| This is a valuable and timely piece of research that adds to our understanding of urgent care utilisation. The suggestions provided are intended to strengthen the paper’s clarity and ensure that its interpretations remain appropriately balanced and transparent. I hope the authors will receive them in the spirit of refining an already high-quality piece of work. | Thank you. |
